# Associations Between Social Network Relationships, Cognitive Functioning, and Financial Exploitation

**DOI:** 10.1093/geroni/igaf122.935

**Published:** 2025-12-31

**Authors:** Gali Weissberger, Shira Peleg, Ella Cohn-Schwartz

**Affiliations:** Bar-Ilan University, Ramat Gan, HaMerkaz, Israel; Bar-Ilan University, Ramat Gan, HaMerkaz, Israel; Ben Gurion University of the Negev, Be’er Sheva, HaDarom, Israel

## Abstract

Financial exploitation (FE) of older adults usually involves a social interaction between the older adult and the exploiter. However, few studies have comprehensively examined social network compositions of older adults who experienced FE. In this study utilizing data from the Survey of Health, Ageing, and Retirement in Europe and Israel (SHARE) Wave 8, we examined whether social relationships, cognitive functioning, and their interactions, are associated with FE in a nationally representative sample of Israeli older adults. Participants (N = 704; M age=71.90, SD = 8.08, Range=53-103) self-reported whether or not they experienced FE over the past 12 months. They also completed social network and cognitive assessments. A global composite score of cognition was calculated based on scores across four cognitive tests. A logistic regression model regressed FE on the presence/absence of children, spouses, other family, and friends in networks, global cognition, and covariates. Interactions between global cognition and relationship types were also tested. The results demonstrated that the presence of children and friends in one’s close network were associated with a lower likelihood of experiencing FE (Children: OR = 0.57, p = 0.011; Friends: OR = 0.30, p < 0.001). Interactions revealed that the association of having friends in the network with FE was significant for those with average or above average global cognition, but not for those with below average global cognition. Findings suggest that friends may play a critical role in protecting against FE when cognition is intact, possibly because family members have less involvement in financial decisions in such circumstances.

